# Exploring Shootin1’s oncogenic role within *FGFR2* gene fusions

**DOI:** 10.55730/1300-0152.2782

**Published:** 2025-09-28

**Authors:** Volkan ERGİN, Mutlu ERDOĞAN, Ekrem YAŞAR, Sika ZHENG

**Affiliations:** 1 Division of Biomedical Sciences, University of California, Riverside, USA; 2 Institute of Biotechnology, Ankara University, Ankara, Turkiye; 3 Department of Biophysics, Faculty of Medicine, Erzincan Binali Yildirim University, Erzincan, Turkiye

**Keywords:** *FGFR2*, *SHTN1*, *KIAA1598*, *FGFR2-SHTN1*, gene fusion, cancer

## Abstract

**Background/aim:**

Fibroblast Growth Factor Receptor (*FGFR*) gene fusions are recognized as pivotal oncogenic drivers, contributing to cancer initiation and progression across diverse malignancies. These fusions often represent significant therapeutic targets, particularly in challenging malignancies like cholangiocarcinoma. This study aimed to characterize the novel *FGFR2*::*SHTN1* fusion, identify it as a de novo chimeric protein, and elucidate its precise oncogenic mechanism.

**Materials and methods:**

*FGFR2*::*SHTN1* fusions were identified via cancer genomics databases and modeled using AlphaFold and HADDOCK. *SHTN1* variants were expressed in Neuro-2a cells for coimmunoprecipitation, purification, and native polyacrylamide gel electrophoresis to assess oligomerization. Structural modeling included membrane embedding with Chemistry at HARvard Macromolecular Mechanics–Graphical User Interface (CHARMM–GUI).

**Results:**

We found that *FGFR2*::*SHTN1* is an in-frame fusion formed by the joining of upstream *FGFR2* exons 1–17 with downstream *SHTN1* exons 7–17 in human, resulting in a chimeric protein retaining the intact *FGFR2* tyrosine kinase domain. Our analyses revealed that Shootin1 inherently forms oligomers through its coiled–coil domains, which, within the fusion, mediate ligand-independent dimerization and constitutive activation of *FGFR2*.

**Conclusion:**

Our findings establish *FGFR2*::*SHTN1* as a potent oncogenic driver in various cancers, particularly in cholangiocarcinoma, highlighting a unique mechanism of constitutive activation mediated by Shootin1’s CCD-II domain. This study represents the first molecular characterization of the *FGFR2*::*SHTN1* fusion, advances understanding of *FGFR2* fusion biology, and identifies a particular target for future diagnostic and therapeutic strategies in relevant malignancies.

## 1. Introduction

*SHTN1* (also known as Shootin1 or *KIAA1598*) is an intriguing protein initially identified for its critical roles in neuronal development. We previously demonstrated that it plays a central role in regulating neuronal polarity and axonogenesis by directly modulating cytoskeletal organization, a function tightly controlled by alternative splicing (Zhang et al., 2019; Ergin and Zheng, 2020).

In an earlier study, we showed that the alternative splicing of Shootin1 pre-mRNA generates a long isoform (SHTN1L) and a short isoform (SHTN1S) (Ergin et al., 2015). While SHTN1S is considered a neuron-specific isoform, SHTN1L is more broadly expressed across various tissues, including the brain (Higashiguchi et al., 2016; Zhang et al., 2019). However, the functional role of the Shootin1 protein in nonneuronal tissues remains poorly understood. In the neuronal context, SHTN1S promotes axon specification by asymmetrically accumulating in a single neurite, whereas the long isoform, SHTN1L, regulates cytoskeletal dynamics to control axon elongation during brain development (Zhang et al., 2019; Zheng, 2020).

Although both isoforms share an identical N-terminal sequence containing coiled–coil domains (CCD), the unique C terminus of SHTN1L grants it a distinct actin-binding property (Zhang et al., 2019; Ergin and Zheng, 2020). The difference in actin binding between the isoforms is a result of the presence or absence of the C-terminal sequence, which modulates the intramolecular interactions of their shared CCD parts. This functional multimodality arises from differential protein structures generated by alternative splicing. Note that, throughout this study, the protein name “Shootin1 or *SHTN1*” specifically refers to the long isoform, SHTN1L, unless otherwise noted.

Interestingly, alongside its function in cytoskeletal reorganization, Shootin1 has recently been implicated in the initiation and progression of various cancers, including bladder, lung, breast, and brain (Uguen and De Braekeleer, 2016; Qin et al., 2019; Li et al., 2020; Nicolò et al., 2023; Morin et al., 2024; Wang et al., 2024). It has been particularly identified and verified in oncogene fusions in cholangiocarcinoma (CCA) tumors (Krook et al., 2020; Li et al., 2020; Neumann et al., 2022; Silverman et al., 2022). The apparent dichotomy in Shootin1’s distinct biological roles suggests that the molecular mechanisms underlying its function may be coopted or dysregulated in oncogenic processes.

While the exact mechanisms underlying Shootin1’s involvement in tumorigenesis remain to be revealed, emerging evidence suggests its involvement in oncogenic gene fusions. In this regard, a significantly prevalent oncogenic partner of Shootin1 in such observed fusions is Fibroblast Growth Factor Receptor 2 (*FGFR2*) (Krook et al., 2020; Li et al., 2020; Silverman et al., 2022; Wang et al., 2024). It is well-established that most *FGFR2* fusion partners promote the constitutive activation of FGF receptor signaling. These fusions typically involve the partner of the *FGFR2* providing specific domains such as coiled–coil, SAM (sterile alpha motif), or caspase that facilitate persistent receptor dimerization, leading to ligand-independent activation of the kinase domain of *FGFR2* (Parker et al., 2014; Tuna et al., 2019; De Luca et al., 2020).

The presence of three consecutive coiled–coil domains within the Shootin1 protein structure inherently suggests the potential for self-association via oligomerization (Ergin and Zheng, 2020). Taken together with the mechanisms governing *FGFR2* oncogenic fusions, wherein coiled–coil domains mediate constitutive receptor dimerization, it is plausible that Shootin1 has the capacity for CCD oligomerization, which could likely be the mechanism driving *FGFR2*’s observed oncogenic activity.

The potential for CCD-mediated oligomerization in Shootin1, coupled with the notable genomic proximity and architecture of the *SHTN1* and *FGFR2* genes on human chromosome 10, suggests a compelling model for the observed propensity of these proteins to participate in oncogenic fusions. We hypothesized that a chromosomal deletion event between *FGFR2* and *SHTN1* could juxtapose the kinase domain of *FGFR2* with the CCD domains of Shootin1. This juxtaposition would cause ligand-independent constitutive activation of *FGFR2* signaling via Shootin1-induced dimerization, thereby driving oncogenesis, particularly in tissues where the *FGFR2* promoter is active. In this study, we propose a mechanistic model to elucidate the plausibility of the *FGFR2*::*Shootin1* oncogene fusion by providing critical evidence for Shootin1 oligomerization and establishing a link between constitutive *FGFR2* activity across various forms of malignancy.

## 2. Materials and methods

### 2.1. Databases and computational tools

Alterations in the *SHTN1* gene across various cancer types were visualized using cBioPortal^1^ (Gao et al., 2013). *FGFR2*::*SHTN1* fusion events were identified using cBioPortal and the COSMIC database^2^ (Tate et al., 2019). Genomic coordinates of in-frame breakpoints in *FGFR2* and *SHTN1* were obtained from the UCSC Genome Browser on the human genome assembly GRCh37/hg19. Exon boundaries for each gene were retrieved from the Consensus CDS (CCDS) database^2^,^3^ (Pruitt et al., 2009). Domain compositions of the wild-type genes and putative fusion products were defined through literature review (Ross et al., 2014; Helsten et al., 2016; Zheng, 2020; Brown et al., 2023). Liver tissue single-cell RNA-seq datasets were accessed via the UCSC Cell Browser interface^4^.

The presence of a coiled–coil domain in Shootin1 was confirmed using the Waggawagga server^5^ (Simm et al., 2014), which employs the ab initio coiled–coil prediction algorithm MARCOIL (Delorenzi and Speed, 2002). The computationally predicted coiled–coil domain II (CCD-II), implicated in oligomerization, was modeled using CCBuilder 2.0^6^ (Wood and Woolfson, 2018). To provide a structural basis for the putative fusion protein, three-dimensional (3D) models were obtained from AlphaFold DB^7^ (Varadi et al., 2024). The Shootin1 structure was further analyzed using QSalign, a homomer prediction tool available through the 3D-Beacons Network (Dey et al., 2018; Varadi et al., 2022). All protein structures were visualized and customized in PyMOL 8.

### 2.2. Recombinant DNA constructs

In this study, pEGFP-C1 and pCAGIG were used as host vectors, as previously described elsewhere (Zhang et al., 2019; Ergin and Zheng, 2020). All constructs were fully sequenced prior to transfection to confirm the absence of mutations introduced during molecular cloning. Plasmids were propagated in *Escherichia coli* DH5α cells and prepared using Qiagen miniprep kits. Recombinant plasmids expressing EGFP-fused *SHTN1* and coiled–coil domain (CCD) deletion variants were generated by cloning the corresponding coding sequences into the XhoI and TspMI (New England Biolabs) sites of the pEGFP-C1 mammalian expression vector. For purification of the target proteins, pCAGIG-based constructs encoding FLAG-tagged Shootin1 were generated by polymerase chain reaction (PCR) amplification of the insert, followed by cloning into the pCAGIG vector linearized with XhoI and NotI (New England Biolabs). Oligonucleotide primer sequences used for cloning into pEGFP-C1 and pCAGIG have been reported previously [Ergin and Zheng, 2020].

### 2.3. Cell culture and transient expression

Neuro-2a cells [*Mus musculus* brain neuroblastoma; American Type Culture Collection (ATCC) #CCL-131] were cultured in Dulbecco’s Modified Eagle Medium (DMEM) supplemented with high glucose, 10% fetal bovine serum (Thermo Fisher Scientific), and two mM GlutaMAX (Thermo Fisher Scientific), following the manufacturer’s instructions. Cells were transfected with expression plasmids, followed by coimmunoprecipitation (co-IP) or protein purification assays. Briefly, Neuro-2a cells were plated at a density of 1 × 10^6^ cells per 10-cm dish and transfected with the indicated plasmids using GeneTran-III (Biomiga), according to the manufacturer’s protocol. Forty-eight hours posttransfection, cells were harvested for downstream applications.

### 2.4. Co-IP and immunoblotting

To assess interactions between FLAG- and EGFP-tagged Shootin1 variants via co-IP, Neuro-2a cells transiently expressing either EGFP or FLAG-fused full-length and truncated constructs were lysed in 1 mL of lysis buffer containing 50 mM Tris-HCl (pH 7.4), 150 mM NaCl, 0.5% Triton X-100, protease/phosphatase inhibitor cocktails (Roche), one mM PMSF, and 100 U/mL Turbonuclease. Cell lysates were incubated with 12 μL GFP-Trap magnetic beads (Chromotek) for one h at 4 °C with gentle rotation. Beads were subsequently washed three times with 1 mL of stringent wash buffer containing 50 mM Tris-HCl (pH 7.4), 300 mM NaCl, 0.5% Triton X-100, and one mM PMSF. Bead–bound proteins were eluted by boiling in 15 μL of SDS sample buffer, and 10 μL of each sample was loaded for sodium dodecyl sulfate–polyacrylamide gel electrophoresis (SDS–PAGE) and immunoblotting with target antibodies. An additional five μL was used to probe for GFP as an expression control. Total protein lysates and immunoprecipitated fractions were analyzed by immunoblotting. Proteins were resolved on polyacrylamide gels, transferred to Immobilon-FL polyvinylidene fluoride (PVDF) membranes, and probed with primary antibodies: chicken polyclonal anti-GFP (1:2500, GFP-1020, AvesLab) and mouse monoclonal anti-FLAG M2 (1:2500, F3165, Sigma-Aldrich). Detection was performed using Alexa Fluor-conjugated secondary antibodies (Thermo Fisher Scientific; 1:2000), and blots were visualized using the GE Typhoon FLA 9000 imaging system.

### 2.5. Protein expression and purification

The pCAGIG-FLAG-SHTN1 construct was individually transfected into Neuro-2a cells cultured in 15-cm dishes. Forty-eight hours posttransfection, cells overexpressing FLAG-tagged *SHTN1* were washed with ice-cold phosphate-buffered saline (PBS), scraped, and lysed in five mL of lysis buffer containing 50 mM Tris-HCl (pH 7.4), 500 mM NaCl, one mM ethylenediaminetetraacetic acid (EDTA), 1% Triton X-100, protease/phosphatase inhibitor cocktails (Roche), one mM phenylmethylsulfonyl fluoride (PMSF), and 100 U/mL Turbonuclease (Sigma-Aldrich). For affinity purification, 200 μL of anti-FLAG M2 magnetic bead slurry (Sigma-Aldrich), preequilibrated in lysis buffer, was added to the cleared lysate and incubated for 3 hours at 4 °C with gentle rotation. Beads were then collected using a magnetic rack and washed three times with 2 mL of lysis buffer. Bound proteins were eluted twice at room temperature for 15 min each using 1 mL of elution buffer containing 10 mM Tris-HCl (pH 7.4), 100 mM NaCl, and 200 μg/mL 3 × FLAG peptide (Apex Bio). Eluted fractions were concentrated and buffer-exchanged into Tris-buffered saline (TBS) using Amicon Ultra centrifugal filters (MWCO 30 kDa; Millipore). Purified FLAG-SHTN1 protein and BSA standards were analyzed by PAGE followed by Coomassie Brilliant Blue R-250 staining (Teknova) to assess purity and estimate the molar concentration of the target protein.

### 2.6. Nondenaturing native PAGE

The gel was prepared using 8% acrylamide/bis-acrylamide (30%/0.8% w/v), 0.375 M Tris-HCl, 10% ammonium persulfate, and 0.1% N,N,N′,N′-Tetramethylethylenediamine (TEMED). Protein samples were brought to 31.2 mM Tris-HCl (pH 8.8), 12.5% (v/v) glycerol, and 0.5% Bromophenol Blue, and were not heat-denatured prior to loading. The running buffer consisted of 25 mM Tris and 192 mM glycine, adjusted to pH 7.5. No SDS or reducing agents were used in any of the native PAGE buffers. Electrophoresis was performed at 200 V for four h on ice. Recombinant α-actinin, a known dimer-forming protein, was used as a positive control and processed identically to the purified *FLAG-SHTN1*. For Coomassie Brilliant Blue R-250 staining, gels were soaked and gently shaken in ready-to-use stain solution for 30 min, followed by destaining in 20% methanol and 7.5% acetic acid in deionized water, with repeated rinsing until the background was well cleared.

### 2.7. Structural modeling of the *FGFR2::SHTN1* fusion complex

To construct the *FGFR2*::*SHTN1* fusion complex model, we first predicted the dimeric structures of *FGFR2* and Shootin1 independently using AlphaFold-Multimer (Evans et al., 2021). *FGFR2* dimerization was modeled based on its full intracellular and transmembrane domains, whereas the Shootin1 dimer model focused on its coiled–coil domains, which mediate oligomerization. Following separate multimer predictions, the best-ranked *FGFR2* and Shootin1 dimers were subjected to protein–protein docking using HADDOCK 2.4 (Honorato et al., 2024). Among the generated docking solutions, the model with the lowest HADDOCK score and highest cluster occupancy was selected for further analysis. To mimic a realistic membrane environment, the selected *FGFR2*::*SHTN1* complex was embedded into a lipid bilayer [1-palmitoyl-2-oleoyl-sn-glycero-3-phosphocholine (POPC)] using the CHARMM-GUI Membrane Builder (Wu et al., 2014). The orientation of the *FGFR2* transmembrane helices was manually refined to match experimental topology. Finally, structural visualization and figure preparation were performed in UCSF ChimeraX (Meng et al., 2023), where the distinct domains of the fusion complex were colored for clarity.

### 2.8. Statistical analysis

Statistical analysis of co-IP experiments performed in three biological replicates was conducted using unpaired Student’s t-tests in GraphPad Prism 10. Data are presented as mean ± SEM. P-values less than 0.05 were considered statistically significant.

## 3. Results

### 3.1. Shootin1 is involved in diverse oncogenic modalities

**Figure 1:**
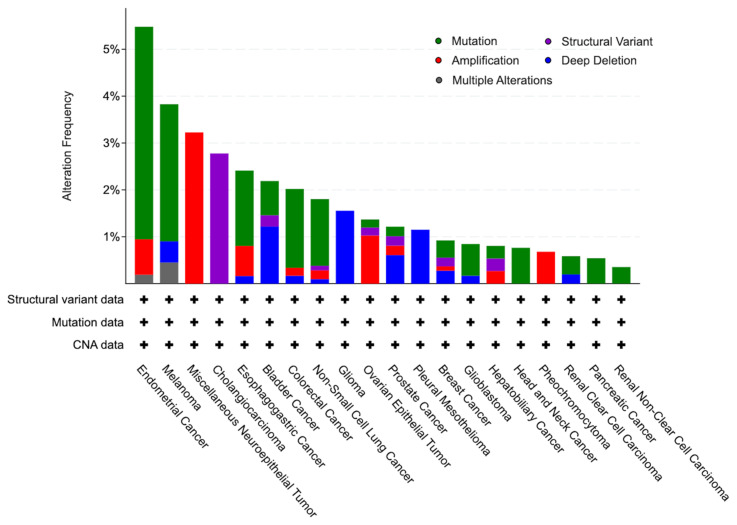
Bar chart summarizing the frequency and types of *SHTN1* gene alterations across various cancer types based on the data from 10,967 patient samples spanning 32 cancer types and 154 individuals in the TCGA PanCancer database.

Our pan-cancer analysis reveals broad dysregulation of *SHTN1*, with distinct alteration patterns across tumor types. We retrieved and visualized *SHTN1* genomic alteration frequencies from The Cancer Genome Atlas (TCGA) Pan-Cancer Atlas studies using cBioPortal. From 10,967 patient samples spanning 32 cancer types in the Atlas, our query identified *SHTN1* alterations in 154 individuals (Figure 1). Despite their presence across numerous cancers, it remains unclear whether these *SHTN1* alterations represent drivers of tumorigenesis or are merely passenger events; their specific molecular correlates thus require elucidation. However, numerous studies have well established that fundamental cancer hallmarks such as cell migration, invasion, chromatin instability, and remodeling involve aberrant cytoskeleton and actin dynamics (Caridi et al., 2019; Izdebska et al., 2020; Datta et al., 2021). Given Shootin1’s roles in regulating cytoskeletal organization and actin dynamics during physiological processes, it is conceivable that *SHTN1* mutations and copy number variations could disrupt these fundamental processes in certain tissues or specific microenvironments, thereby contributing to the malignant phenotype.

Shootin1’s diverse alterations indicate an intertwined, multifaceted involvement in cancer, ranging from mere presence to mechanistic collaboration. A detailed analysis of *SHTN1* alterations in the cBioPortal and COSMIC databases enabled us to curate a table highlighting specific *SHTN1* gene fusions, including those with reported oncogenic activity (Table). The data reveal a variety of *SHTN1* fusions; however, the intrachromosomal *FGFR2*::*SHTN1* and interchromosomal *ROS1*::*SHTN1* fusions stand out for their well-annotated and experimentally verified roles as drivers of oncogenesis (Ross et al., 2014; Wiesner et al., 2014). Notably, both *FGFR2* and *ROS1* belong to the family of Receptor Tyrosine Kinases (RTKs), critical regulators of intracellular signaling pathways involved in cell growth and differentiation. Aberrant activation of such RTKs, often through gene fusions, is a well-established mechanism in oncogenesis, typically leading to constitutive kinase activity that drives uncontrolled cell proliferation. In both fusion types, *SHTN1* acts as the tail gene (the 3’ partner in the fusion), resulting in an in-frame final construct. This in-frame designation indicates that the fusion retains an open reading frame within the chimeric protein, allowing the fused gene products to be translated without premature termination. The fact that *SHTN1* is the tail gene suggests that the kinase domains of *ROS1* and *FGFR2* are potentially driving the oncogenic activity, whereas Shootin1 likely promotes the dimerization of these kinase domains.

While both *ROS1*::*SHTN1* and *FGFR2*::*SHTN1* fusions represent intriguing oncogenic events involving Shootin1, we will primarily focus on the *FGFR2*::*SHTN1* fusion in this study. This is largely due to extensive literature confirming this fusion as potent and often targetable in malignancies, which also provides a rich context for mechanistic understanding. Thus, *FGFR2*::*SHTN1* is a promising, biologically plausible candidate for developing a detailed model of Shootin1’s role in oncogenesis.

### 3.2. The interplay of Shootin1 and *FGFR2* in cancer

*FGFR2*, as a pivotal RTK, normally plays critical roles in regulating cell proliferation, differentiation, and survival through tightly controlled ligand-dependent signaling. Gene fusions involving *FGFR2* are well-established oncogenic drivers across numerous cancer types. These fusions typically arise from chromosomal rearrangements that juxtapose the intact kinase domain of *FGFR2* with a dimerization or oligomerization domain from a partner gene, which eliminates the need for ligand binding, resulting in constitutive activation of *FGFR2*’s kinase activity and subsequent aberrant signaling through downstream pathways like MAPK/ERK and PI3K/Akt (Gallo et al., 2015). Such sustained activation drives uncontrolled cell growth and migration, thereby conferring significant oncogenic potential to *FGFR2* fusions, which have led to their recognition as clinical targets (Parker et al., 2014; Borad et al., 2015; De Luca et al., 2020).

In this regard, CCA exhibits particularly high FGFR fusion prevalence and therapeutic potential, and importantly, *FGFR2* is emerging as a significant fusion partner, along with its associate protein Shootin1 (Krook et al., 2020; Li et al., 2020; Deng et al., 2023).

**Figure 2:**
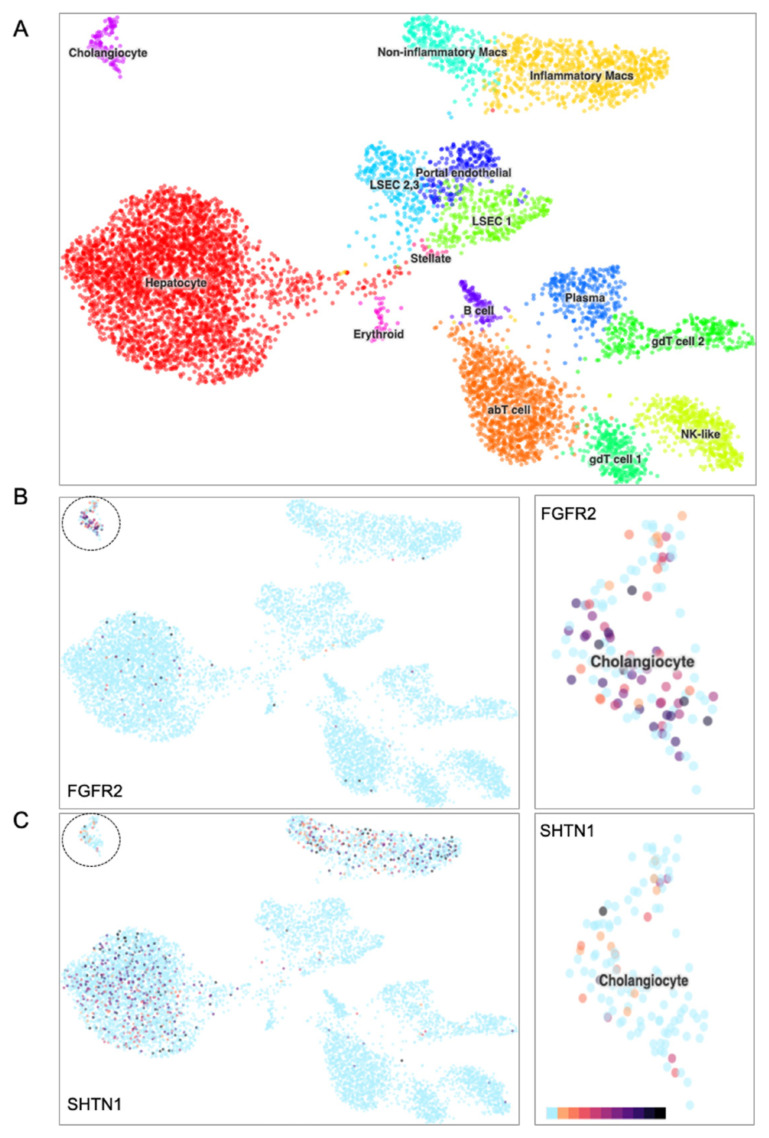
Single-cell RNA sequencing of human liver reveals enriched *FGFR2* expression and the absence of *SHTN1* in cholangiocytes. A) Cluster map showing assigned cell identities in a *t*-SNE plot of 8,444 liver cells, where cells with similar transcriptomic profiles are grouped by color based on unsupervised clustering. B–C) *t*-SNE projections illustrating the expression patterns of *FGFR2* and *SHTN1* in liver cells, along with corresponding close-up views highlighting gene expression within the cholangiocyte population. The expression of marker genes SOX9, EPCAM, and KRT19 identifies cholangiocytes. The color scale ranges from cyan (no expression) to dark purple (high expression) for the genes of interest.

CCA is a highly lethal malignancy that arises predominantly from the epithelial cells lining the bile ducts, known as cholangiocytes (Brindley et al., 2021). Given the critical role of cholangiocytes in CCA pathogenesis and the experimentally validated oncogenicity of the *FGFR2*::*SHTN1* fusion in CCA, we investigated baseline expression of both genes in healthy human cholangiocytes using publicly available single-cell RNA sequencing data from deceased donor liver cells (MacParland et al., 2018; Speir et al., 2021). Our analysis of a t-SNE plot showing various liver cell identities revealed distinct expression patterns for *FGFR2* and *SHTN1* within the cholangiocyte population (Figure 2A). While *FGFR2* showed robust and widespread expression across healthy cholangiocytes (Figure 2B), *SHTN1* expression was notably absent within these same cells (Figure 2C). This differential expression suggests that the *FGFR2*::*SHTN1* fusion observed in CCA indicates the presence of a de novo chimeric protein in cells that normally lack the Shootin1 component. Thus, we anticipate that this novel chimeric transcript is expressed under the control of the upstream *FGFR2* promoter, given that *FGFR2* serves as the 5′ fusion partner. Yet, through single-cell RNA sequencing of patient samples, further validation would fully confirm this anticipated cellular context. In this regard, we would expect single-cell data sampled from CCA biopsies to show Shootin1 as detectable, whereas *FGFR2* may appear absent. This is because most single-cell RNA-seq platforms rely on polyadenylic acid capture and sequence only a short stretch from the 3′ end. Since *FGFR2* is located further upstream in the fusion transcript, it may lie outside the sequencing read window and therefore go undetected. At the same time, *SHTN1*, positioned at the immediate 3′ end, would be captured and sequenced.

**Figure 3:**
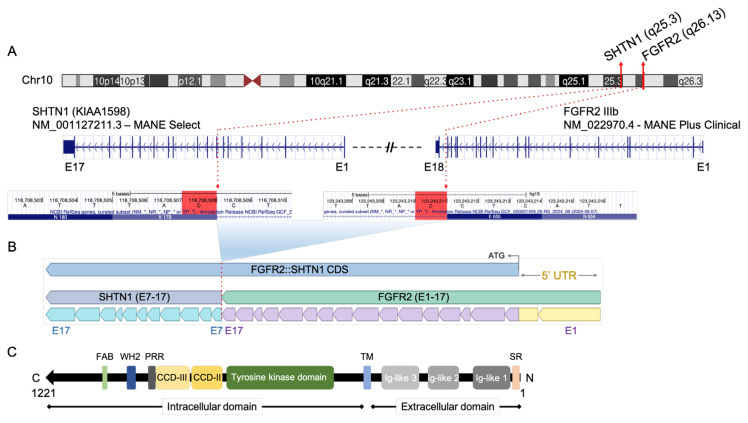
Genetic architecture of *FGFR2*::*SHTN1* fusion. A) Schematic representations of the *FGFR2*::*SHTN1* fusion identified in human cancers, showing the in-frame breakpoint at chromosomal, gene, and nucleotide resolutions. B) *FGFR2*::*SHTN1* fusion product with *FGFR2* exons 1–17 at the N-terminus fused to the C-terminal *SHTN1*’s exons 7–17. C) Chimeric protein structure, retaining an intact tyrosine kinase domain, fused to the C-terminal Shootin1. SR: signal peptide region, TM: transmembrane domain, CCD: coiled–coil domain, PRR: proline-rich region, WH2: WASP homology domain-2, FAB: F-actin binding domain.

The *FGFR2* and *SHTN1* genes are in proximity on human chromosome 10, with FGFR2 upstream of SHTN1. The precise genetic architecture of the *FGFR2*::*SHTN1* fusion identified in human cancers is schematically represented in Figure 3A. With the critical in-frame breakpoints, the fusion results from the joining of *FGFR2* exons 1–17 to the C-terminal portion of *SHTN1*, encompassing its exons 7–17, thereby ensuring a continuous coding sequence (Figure 3B). Crucially, this chimeric construct retains the entire intracellular tyrosine kinase domain, a fundamental structural arrangement that positions the potent *FGFR2* kinase for ligand-independent activation and oncogenic activity.

The constitutive activation conferred by the *FGFR2* kinase, thus the unique oncogenic potential of *FGFR2*::*SHTN1*, is likely modulated by the incorporated C-terminal domains of Shootin1. The fusion protein specifically includes Shootin1’s coiled–coil domains (CCD-II and CCD-III) (Figure 3C). The presence of the coiled–coil domain suggests a mechanism for ligand-independent dimerization driving the constitutive activity of the fused *FGFR2* kinase. This intricate molecular architecture suggests that Shootin1 possesses an inherent capacity to oligomerize via its coiled–coil domains, enabling constitutive and aberrant signaling by the oncogenic fusion protein within tumor cells.

### 3.3. Shootin1 oligomerizes through its coiled–coil domains

**Figure 4:**
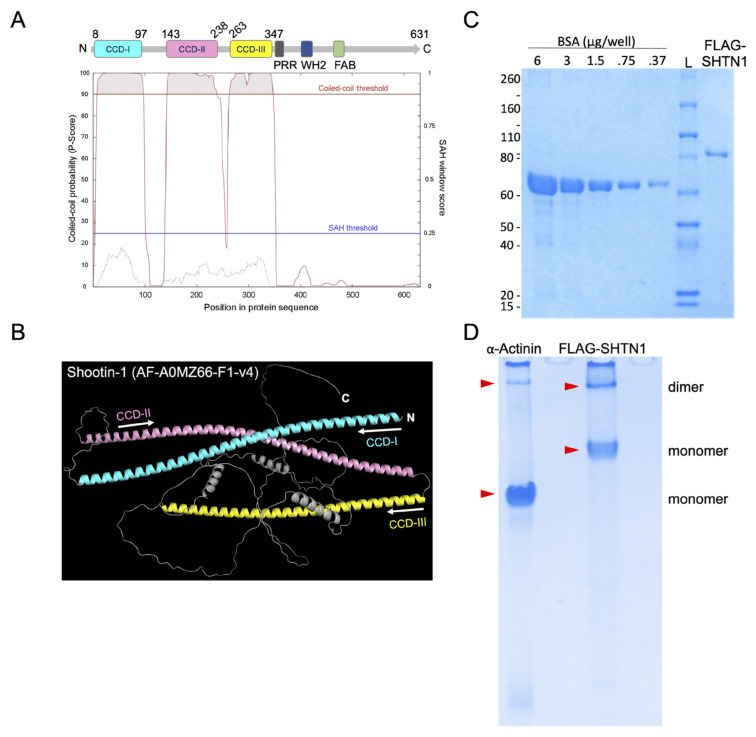
Shootin1 contains three consecutive coiled–coil domains. A) The coiled–coil profile of Shootin1 predicted by Marcoil. The vertical scale shows the coiled–coil domain probability (0–100), and the horizontal scale shows amino acid numbers. Regions with high coiled–coil probability scores (>90%) and a 21-residue window are classified as coiled–coil domains (CCD). B) AlphaFold-predicted structure of Shootin1, revealing three distinct coiled–coil domains consistent with Marcoil predictions. C) Coomassie blue-stained SDS–PAGE gel showing the purity of denatured FLAG-Shootin1 protein with no detectable copurified contaminant. The protein concentration was determined by densitometric analysis relative to 1:2 serial dilutions of recombinant BSA, starting at six μg/well with a 10 μL loading volume. D) Native PAGE (8%; pH 7.5) analysis of FLAG-Shootin1 protein under nonreducing conditions, indicating the oligomer state of Shootin1. α-Actinin, a known dimer-forming protein, was used as a positive control.

To investigate the potential role of Shootin1’s coiled–coil domains in the dimerization of the *FGFR2*::*SHTN1* fusion, we first characterized the CCD profile of native Shootin1. In silico prediction using Marcoil (Simm et al., 2014) identified three distinct regions within the Shootin1 sequence exhibiting high coiled–coil probability scores, consistently above 90% (Figure 4A). This prediction was further supported by AlphaFold modeling, which revealed a three-dimensional structure of Shootin1 consistent with the presence of three CCDs (Figure 4B). To investigate whether Shootin1 can form oligomers, we first purified FLAG-tagged Shootin1 protein from mammalian cells. Coomassie staining of the purified samples confirmed the absence of copurifying proteins, indicating high purity of the FLAG-SHTN1 preparation (Figure 4C). We then subjected the samples to native-PAGE under nonreducing conditions. This analysis revealed that FLAG-Shootin1 migrates as a higher-order complex, consistent with an oligomeric state. As a positive control, the well-characterized dimeric protein α-Actinin exhibited a similar migration pattern, supporting the conclusion that Shootin1 oligomerizes under native conditions (Figure 4D). These findings collectively establish that Shootin1 contains three consecutive CCDs and possesses the intrinsic ability to form oligomers.

**Figure 5:**
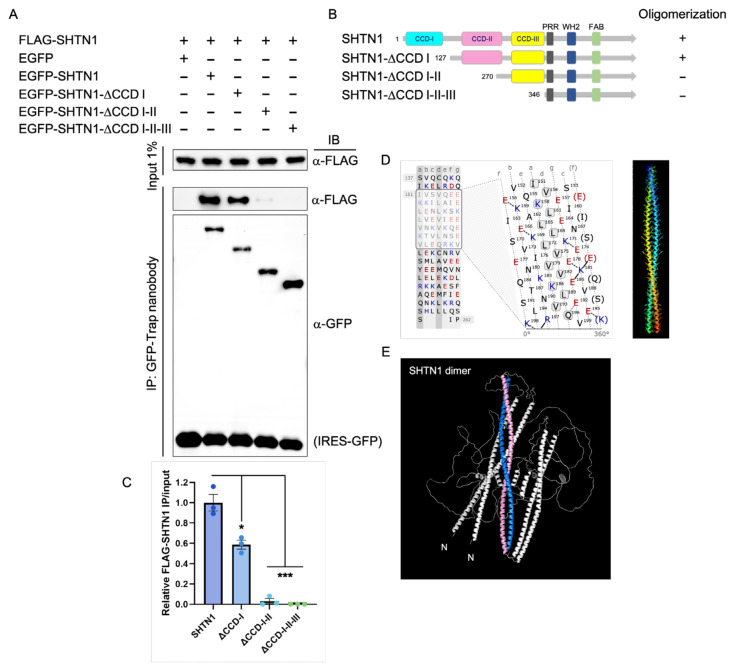
Shootin1 oligomerizes through CCD-II. A) Co-IP experiments of FLAG-fused Shootin1 with EGFP-Shootin1 or EGFP-Shootin1-ΔCCD revealed that Shootin1 forms oligomers in a CCD-II-dependent manner. B) Graphic representation of EGFP-fused constructs used for co-IP experiments. C) The relative ratios of precipitated FLAG-SHTN1 to input FLAG-SHTN1 were calculated (mean ± SEM, n = 3) for each EGFP-fused variant, with the mean ratio for full-length EGFP-SHTN1 arbitrarily set to 1. Asterisks indicate statistically significant decreases in FLAG-SHTN1 pulldown by CCD-deletion constructs compared to full-length EGFP-SHTN1, as determined by unpaired *t*-tests. Exact p-values of 0.011 and 0.0003 are considered statistically significant and are denoted as * and ***, respectively, in the bar graph. D) Left: In silico MARCOIL prediction of the heptad repeat regions in CCD-II. Positions within the heptad repeat are labeled a–g at the top. The occurrence of isoleucine or leucine residues at a and d positions, which favors the formation of oligomers, is shaded in grey. Right: The putative CCD-II region is modelled in CCBuilder to predict the preferred oligomer state. E). The dimerization model of Shootin1 was predicted using the ab initio modeling tool QSalign. Regions of the Shootin1 CCD-II that contribute to oligomerization are colored for visual clarity. N: NH_2_ terminus.

Further investigations were conducted to pinpoint the specific coiled–coil domain responsible for Shootin1’s oligomerization. We coexpressed EGFP- and FLAG-tagged Shootin1 in Neuro-2a cells and tested whether the two protein variants interacted by co-IP. Full-length EGFP-Shootin1 proteins were immunoprecipitated from cell lysates, and immunoblotting was performed to probe FLAG-tagged Shootin1 proteins. This experiment showed that the two protein variants interacted, indicating that the full-length Shootin1 molecules could form oligomers. To test whether CCD regions mediate the oligomerization, we replaced EGFP-SHTN1 with EGFP-tagged CCD deletion mutants in the co-IP experiment. Intermolecular interaction was preserved in the absence of CCD-I, but deletion of CCD-I-II (or CCD-I-II-III) completely abolished the interaction (Figure 5A–C). Since CCD-I alone is not necessary for Shootin1 oligomerization, the CCD-II region likely plays an essential role in proper oligomerization. This experimental validation was supported by in silico analyses of CCD-II. Marcoil prediction of the heptad repeat regions (Delorenzi and Speed, 2002; Simm et al., 2014) within CCD-II showed a pattern that specifically highlights the high occurrence of isoleucine or leucine residues at the ‘a and ‘d positions, which are known to promote oligomer formation (Figure 5D). Furthermore, computational modeling using CCBuilder (Wood and Woolfson, 2018) and QSalign (Dey et al., 2018; Varadi et al., 2024) predicted a preferred dimerization state for the putative CCD-II region, consistent with our experimental observations (Figure 5D and 5E). Given that the *FGFR2*::*SHTN1* fusion construct incorporates the Shootin1 CCD-II region, these results strongly suggest that CCD-II is the primary motif driving the ligand-independent dimerization and subsequent constitutive activation of the *FGFR2* kinase domain within the oncogenic *FGFR2*::*SHTN1* chimeric protein.

### 3.4. Proposed model for *FGFR2::SHTN1* fusion-driven oncogenic activity

**Figure 6:**
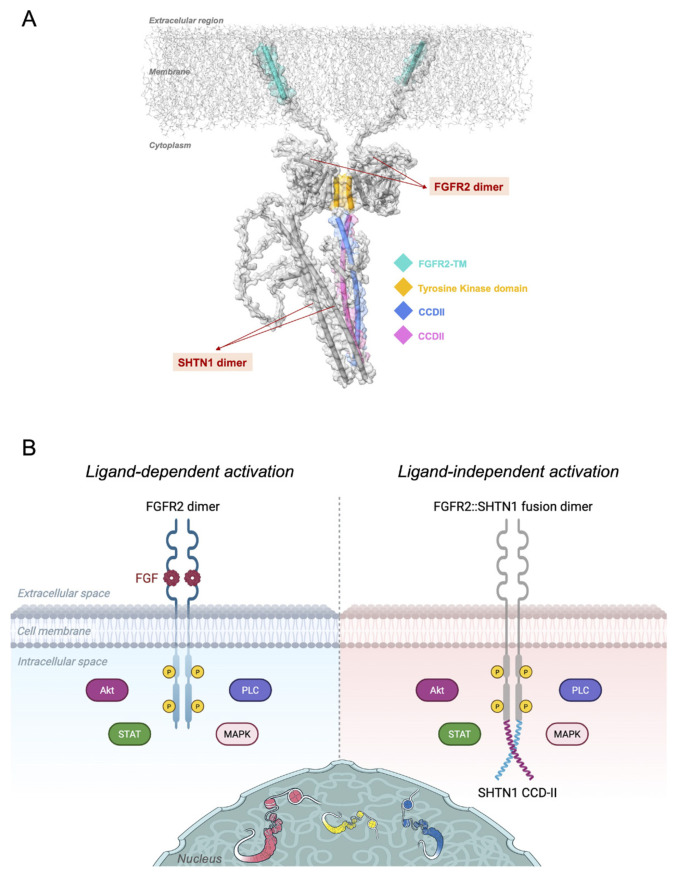
Proposed model for Shootin1 fusion-driven constitutive activation of *FGFR2* signaling in cancer. A) A structural model of the *FGFR2*::*SHTN1* fusion dimer embedded in a lipid bilayer. The *FGFR2* transmembrane helices (*FGFR2*-TM) are shown in cyan, while the tyrosine kinase domains are highlighted in orange, indicating dimerization and signaling competency. The Shootin1 dimer is visualized interacting with the cytoplasmic portion of the *FGFR2* dimer, contributing structural scaffolding and potential downstream signaling effects. CCD-II regions of Shootin1 dimer are colored blue and magenta, individually. This model illustrates the molecular architecture and spatial organization of the fusion construct, shedding light on possible signaling mechanisms initiated by the *FGFR2*::*SHTN1* fusion at the membrane interface. B) Illustration of Shootin1 fusion-driven constitutive activation of *FGFR2* signaling in cancer. Left: In the physiological state, *FGFR2* activation is ligand-dependent, triggered by binding of FGF2, which induces receptor dimerization. This dimerization activates the intracellular tyrosine kinase domains, initiating phosphorylation events that drive downstream signaling cascades and regulate genes involved in cell proliferation, differentiation, and survival. Right: In the oncogenic state, chromosomal rearrangements result in fusion proteins such as *FGFR2*::*SHTN1*, where the N-terminal *FGFR2* portion retains an intact tyrosine kinase domain and is fused to a C-terminal partner Shootin1 that contributes a coiled–coil dimerization domain and promotes ligand-independent dimerization and constitutive autophosphorylation of the *FGFR2* kinase domain.

The detailed characterization of the *FGFR2*::*SHTN1* fusion’s genetic and protein architecture, combined with our findings on Shootin1’s intrinsic oligomerization capabilities, allowed us to propose a comprehensive model for its constitutive activation in cancer (Figure 6A). In its physiological state (Figure 6B, left panel), *FGFR2* activation is tightly regulated and ligand-dependent. The binding of specific FGF ligands induces receptor dimerization, which in turn activates the intracellular tyrosine kinase domains. This initiates a cascade of autophosphorylation events that propagate through the downstream signaling pathways, including MAPK, STAT, AKT, and PLCγ. This controlled signaling is essential for regulating genes involved in normal cell proliferation, differentiation, and survival.

However, in the oncogenic state driven by the *FGFR2*::*SHTN1* fusion (Figure 6B, right panel), this precise and strict regulation control is disrupted. As demonstrated by our structural and functional analyses, the *FGFR2*::*SHTN1* fusion protein retains the *FGFR2* tyrosine kinase domain intact, fused to the C-terminal portion of Shootin1, which contributes the potent coiled–coil dimerization domain CCD-II. This structural arrangement promotes ligand-independent dimerization and subsequent constitutive autophosphorylation of the *FGFR2* kinase domain in the intracellular space. Such aberrant, uncontrolled activation of *FGFR2* signaling then constantly drives downstream oncogenic pathways without the necessary regulatory brakes. This sustained constitutive signaling contributes significantly to malignant progression, including increased cell proliferation, enhanced invasiveness, epithelial–mesenchymal transition, and angiogenesis, which underscores the role of *FGFR2*::*SHTN1* as a potent driver of oncogenesis.

## 4. Discussion

The identification and detailed characterization of the *FGFR2*::*SHTN1* fusion contribute significantly to the growing understanding of oncogenic gene fusions as potent drivers in human cancer. As a family of RTKs, FGFRs are normally vital regulators of cellular proliferation, differentiation, and tissue homeostasis, but their aberrant activation frequently underpins various malignancies.

Gene fusions, in particular, represent a well-established paradigm of oncogenic drivers, and our analyses illuminate the molecular mechanism of the *FGFR2*::*SHTN1* fusion, identifying it as a de novo oncogenic driver, particularly relevant to CCA. The precise genetic architecture, characterized by an in-frame breakpoint between upstream *FGFR2* (exons 1–17) and downstream *SHTN1* (exons 7–17) on human chromosome 10, highlights the critical role of the genomic architecture in facilitating this recurrent rearrangement. This specific configuration preserves the intact *FGFR2* tyrosine kinase domain, with its robust expression likely driven by the native *FGFR2* promoter. Our findings critically demonstrate that Shootin1 possesses intrinsic oligomerization capabilities, specifically mediated by its CCD-II domain. Our findings provide, for the first time, a direct molecular mechanism for the constitutive, ligand-independent dimerization and subsequent autophosphorylation of the fused *FGFR2* kinase through the contribution of the coiled–coil domains of Shootin1 protein. The absence of *SHTN1* expression in healthy cholangiocytes, in contrast to the physiological presence of *FGFR2*, underscores how this fusion creates an entirely novel chimeric protein with aberrant signaling potential in the cells critical for CCA initiation and progression.

While the constitutive activation of the *FGFR2* kinase through Shootin1-mediated dimerization is a clear oncogenic mechanism, the unique presence of Shootin1’s actin-modulating domains (i.e., PRR, WH2, FAB) within the chimeric protein opens avenues for discussions regarding its expanded role in cancer progression. Beyond simply providing a dimerization scaffold, could the fused Shootin1 domains actively reprogram cellular architecture and dynamics? It is conceivable that the activated *FGFR2* kinase might aberrantly regulate components of the actin cytoskeleton directly via its proximity to Shootin1’s domains, or conversely, that the cytoskeletal activities of the fused Shootin1 could feed back into and dysregulate *FGFR2* signaling in novel ways. This could extend beyond increased proliferation to dramatically enhanced cell motility and invasiveness, through unique mechanobiological pathways. Another noteworthy possibility is that the altered cytoskeletal dynamics, driven by this fusion, may generate novel physical forces within the cell that influence drug resistance pathways.

Our previous studies demonstrated that Shootin1 is subject to partial autoinhibition mediated by its N-terminal CCD-I, which attenuates its ability to bind F-actin and may regulate its conformational dynamics (Ergin and Zheng, 2020). In addition to its role in actin regulation, CCD-I also contributes to the cytoplasmic retention of Shootin1 by masking a strong nuclear localization signal (NLS) embedded between the proline-rich region (PRR) and the WH2 domain. Deletion of the coiled–coil domains unmasks the NLS and results in robust nuclear accumulation of Shootin1, highlighting a dual regulatory role for CCD-I in both subcellular localization and cytoskeletal interactions. These regulatory mechanisms become particularly relevant in the context of the *FGFR2*::*SHTN1* fusion identified in cancers. The fusion eliminates the N-terminal CCD-I of Shootin1, potentially releasing both autoinhibitory constraints and the NLS. As a consequence, the chimeric protein may acquire novel biochemical properties, including enhanced actin-binding activity and altered subcellular distribution. Notably, the unmasked NLS from Shootin1 could facilitate nuclear translocation of the *FGFR2* moiety. A hypothesis supported by recent studies links the nuclear localization of *FGFR2* to its emerging role in transcriptional regulation (Lee et al., 2019; Chen et al., 2020; Servetto et al., 2021). Therefore, the *FGFR2*::*SHTN1* fusion may not only affect the cytoskeletal architecture through gain-of-function activity via the truncated Shootin1 protein, but also may confer nuclear functions on *FGFR2*, thus bridging the cytoskeletal remodeling with nuclear signaling in oncogenesis. Moreover, despite the absence of the CCD-I, the Shootin1 portion of the chimeric protein retains its full set of actin-related domains, including PRR, WH2, and the F-actin-binding domain. It is highly plausible that, while the retained CCD-II domain of *FGFR2*::*SHTN1* facilitates ligand-independent receptor activation, the intact C-terminal domains of Shootin1 independently mediate cytoskeletal rearrangements. Together, these properties suggest that the *FGFR2*::*SHTN1* fusion protein may act as a multifunctional oncoprotein, simultaneously driving transcriptional and cytoskeletal programs that promote tumor progression. These unique structural and functional characteristics of *FGFR2*::*SHTN1* not only solidify its standing as a potent and targetable oncogene but also warrant further exploration of its distinct impact on cellular mechanotransduction and its potential as a highly specific diagnostic marker or a multimodal therapeutic target beyond mere kinase inhibition.

## Figures and Tables

**Table:** Manually curated *SHTN1* fusions from public databases, summarizing cancer types, sample IDs, and relevant annotations.

Database	Sample ID	Cancer type	Fusion gene	Head gene	Tail gene	Annotation	Transcript
cBioPortal	TCGA-W5-AA2Q-01	Intrahepatic cholangiocarcinoma	*FGFR2*::*SHTN1*	*FGFR2*	*SHTN1*	Oncogenic	In-frame
	TCGA-5B-A90C-01	Uterine endometrioid carcinoma	*FGFR2*::*SHTN1*	*FGFR2*	*SHTN1*	Oncogenic	In-frame
	TCGA-A8-A094-01	Breast invasive ductal carcinoma	*SHTN1::EMX2OS*	*SHTN1*	*EMX2OS*	Unknown	N/A
	TCGA-LL-A73Z-01	Breast invasive ductal carcinoma	*SHTN1::FAM24B*	*SHTN1*	*FAM24B*	Unknown	N/A
	TCGA-DK-A1A7-01	Bladder urothelial carcinoma	*SHTN1::PRDX3*	*SHTN1*	*PRDX3*	Unknown	N/A
	TCGA-24-1556-01	Serous ovarian cancer	*SHTN1::YTHDF1*	*SHTN1*	*YTHDF1*	Unknown	N/A
	TCGA-CC-5263-01	Hepatocellular carcinoma	*SHTN1::PDZD8*	*SHTN1*	*PDZD8*	Unknown	N/A
	TCGA-EJ-8469-01	Prostate adenocarcinoma	*SHTN1::DZANK1*	*SHTN1*	*DZANK1*	Unknown	N/A
	TCGA-O2-A52V-01	Lung squamous cell carcinoma	*ACIN1::SHTN1*	*ACIN1*	*SHTN1*	Unknown	N/A
	TCGA-Z2-AA3S-06	Cutaneous melanoma	*SHTN1::HSPA12A*	*SHTN1*	*HSPA12A*	Unknown	N/A
COSMIC	COSS2158596	Atypical spitzoid tumor	*ROS1*::*SHTN1*	*ROS1*	*SHTN1*	Oncogenic	In-frame

## Footnotes

1 cBioPortal (2013). TCGA Pan-Cancer Atlas [online]. Website https://www.cbioportal.org [accessed 02 June 2025].

2 COSMIC (2017). Somatic mutations [online]. Website https://cancer.sanger.ac.uk/cosmic [accessed 26 June 2025].

3 CCDS (2009). Consensus CDS [online]. Website https://www.ncbi.nlm.nih.gov/CCDS/CcdsBrowse.cgi [accessed 11 May 2025].

4 UCSC Cell Browser (2021). Single cell data [online]. Website https://human-liver.cells.ucsc.edu [accessed 01 February 2025].

5 Waggawagga (2014). Coiled-coil predictions [online]. Website https://waggawagga.motorprotein.de [accessed 08 January 2025].

6 CCBuilder2.0 (2018). Coiled-coil modeling [online]. Website http://coiledcoils.chm.bris.ac.uk/ccbuilder2/builder [accessed 08 January 2025].

7 AlphaFold DB (2021). Protein structure database [online]. Website https://alphafold.ebi.ac.uk [accessed 05 January 2025].

## References

[b1-tjb-49-07-811] Borad MJ Gores GJ Roberts LR 2015 Fibroblast growth factor receptor two fusions as a target for treating cholangiocarcinoma Current Opinion in Gastroenterology 31 3 264 8 10.1097/MOG.0000000000000171 25763789 PMC4750878

[b2-tjb-49-07-811] Brindley PJ Bachini M Ilyas SI Shahid AK Loukas A 2021 Cholangiocarcinoma Nature Reviews Disease Primers 7 1 65 10.1038/s41572-021-00300-2 PMC9246479 34504109

[b3-tjb-49-07-811] Brown LM Ekert PG Fleuren ED 2023 Biological and clinical implications of FGFR aberrations in paediatric and young adult cancers Oncogene 42 23 1875 88 10.1038/s41388-023-02705-7 37130917 PMC10244177

[b4-tjb-49-07-811] Caridi CP Plessner M Grosse R Chiolo I 2019 Nuclear actin filaments in DNA repair dynamics Nature Cell Biology 21 9 1068 77 10.1038/s41556-019-0379-1 31481797 PMC6736642

[b5-tjb-49-07-811] Chen MK Hsu JL Hung MC 2020 Nuclear receptor tyrosine kinase transport and functions in cancer Advances in Cancer Research 147 59 107 10.1016/bs.acr.2020.04.010 32593407

[b6-tjb-49-07-811] Datta A Deng S Gopal V Yap KCH Halim CE 2021 Cytoskeletal dynamics in epithelial-mesenchymal transition: insights into therapeutic targets for cancer metastasis Cancers 13 8 1882 10.3390/cancers13081882 PMC8070945 33919917

[b7-tjb-49-07-811] De Luca A Esposito Abate R Rachiglio AM Maiello MR Esposito C 2020 FGFR fusions in cancer: from diagnostic approaches to therapeutic intervention International Journal of Molecular Sciences 21 18 6856 10.3390/ijms21186856 32962091 PMC7555921

[b8-tjb-49-07-811] Delorenzi M Speed T 2002 An HMM model for coiled-coil domains and a comparison with PSSM-based predictions Bioinformatics 18 4 617 25 10.1093/bioinformatics/18.4.617 12016059

[b9-tjb-49-07-811] Deng M Ran P Chen L Wang Y Yu Z 2023 Proteogenomic characterization of cholangiocarcinoma Hepatology 77 2 411 29 10.1002/hep.32624 35716043 PMC9869950

[b10-tjb-49-07-811] Dey S Ritchie DW Levy ED 2018 PDB-wide identification of biological assemblies from conserved quaternary structure geometry Nature Methods 15 1 67 72 10.1038/nmeth.4510 29155427

[b11-tjb-49-07-811] Ergin V Erdogan M Menevse A 2015 Regulation of Shootin1 gene expression involves NGF-induced alternative splicing during neuronal differentiation of PC12 cells Scientific Reports 5 1 17931 10.1038/srep17931 26648138 PMC4673418

[b12-tjb-49-07-811] Ergin V Zheng S 2020 Putative coiled-coil domain-dependent autoinhibition and alternative splicing determine SHTN1’s actin-binding activity Journal of Molecular Biology 432 14 4154 66 10.1016/j.jmb.2020.04.025 32371045 PMC7418779

[b13-tjb-49-07-811] Evans R O’Neill M Pritzel A Antropova N Senior A 2021 Protein complex prediction with AlphaFold-Multimer bioRxiv 4 2021 10 10.1101/2021.10.04.463034

[b14-tjb-49-07-811] Gallo LH Nelson KN Meyer AN Donoghue DJ 2015 Functions of Fibroblast Growth Factor Receptors in cancer defined by novel translocations and mutations Cytokine & Growth Factor Reviews 26 4 425 49 10.1016/j.cytogfr.2015.03.003 26003532

[b15-tjb-49-07-811] Gao J Aksoy BA Dogrusoz U Dresdner G Gross B 2013 Integrative analysis of complex cancer genomics and clinical profiles using the cBioPortal Science Signaling 6 269 pl1 10.1126/scisignal.2004088 23550210 PMC4160307

[b16-tjb-49-07-811] Helsten T Elkin S Arthur E Tomson BN Carter J 2016 The FGFR landscape in cancer: Analysis of 4,853 tumors by next-generation sequencing Clinical Cancer Research 22 1 259 67 10.1158/1078-0432.CCR-14-3212 26373574

[b17-tjb-49-07-811] Higashiguchi Y Katsuta K Minegishi T Yonemura S Urasaki A 2016 Identification of a shootin1 isoform expressed in peripheral tissues Cell and Tissue Research 366 1 75 87 10.1007/s00441-016-2415-9 27177867

[b18-tjb-49-07-811] Honorato RV Trellet ME Jiménez-García B Schaarschmidt JJ Giulini M 2024 The HADDOCK2. 4 web server for integrative modeling of biomolecular complexes Nature Protocols 19 11 3219 41 10.1038/s41596-024-01011-0 38886530

[b19-tjb-49-07-811] Izdebska M Zielińska W Hałas-Wiśniewska M Grzanka A 2020 Involvement of actin and actin-binding proteins in carcinogenesis Cells 9 10 2245 10.3390/cells9102245 33036298 PMC7600575

[b20-tjb-49-07-811] Krook MA Lenyo A Wilberding M Barker HD Dantuono M 2020 Efficacy of FGFR inhibitors and combination therapies for acquired resistance in FGFR2-fusion cholangiocarcinoma Molecular Cancer Therapeutics 19 3 847 57 10.1158/1535-7163.MCT-19-0631 31911531 PMC7359896

[b21-tjb-49-07-811] Lee JE Shin SH Shin HW Chun YS Park JW 2019 Nuclear FGFR2 negatively regulates hypoxia-induced cell invasion in prostate cancer by interacting with HIF-1 and HIF-2 Scientific Reports 9 1 3480 10.1038/s41598-019-39843-6 30837551 PMC6401139

[b22-tjb-49-07-811] Li F Peiris MN Donoghue DJ 2020 Functions of FGFR2 corrupted by translocations in intrahepatic cholangiocarcinoma Cytokine & Growth Factor Reviews 52 56 67 10.1016/j.cytogfr.2019.12.005 31899106

[b23-tjb-49-07-811] MacParland SA Liu JC Ma XZ Innes BT Bartczak AM 2018 Single cell RNA sequencing of human liver reveals distinct intrahepatic macrophage populations Nature Communications 9 1 4383 10.1038/s41467-018-06318-7 PMC6197289 30348985

[b24-tjb-49-07-811] Meng EC Goddard TD Pettersen EF Couch GS Pearson ZJ 2023 UCSF ChimeraX: Tools for structure building and analysis Protein Science 32 11 e4792 10.1002/pro.4792 37774136 PMC10588335

[b25-tjb-49-07-811] Morin E Apfelbaum AA Sturm D Ayoub G DiGiacomo J 2024 A diverse landscape of FGFR alterations and co-mutations defines novel therapeutic strategies in pediatric low-grade gliomas bioRxiv 28 2024 08 10.1101/2024.08.27.609922

[b26-tjb-49-07-811] Neumann O Burn TC Allgäuer M Ball M Kirchner M 2022 Genomic architecture of FGFR2 fusions in cholangiocarcinoma and its implication for molecular testing British Journal of Cancer 127 8 1540 9 10.1038/s41416-022-01908-1 35871236 PMC9553883

[b27-tjb-49-07-811] Nicolò E Munoz-Arcos L Vagia E Reduzzi C Donahue J 2023 Circulating tumor DNA and unique actionable genomic alterations in the longitudinal monitoring of metastatic breast cancer: A case of FGFR2-kiaa1598 gene fusion JCO Precision Oncology 7 e2200702 10.1200/PO.22.00702 37437229

[b28-tjb-49-07-811] Parker BC Engels M Annala M Zhang W 2014 Emergence of FGFR family gene fusions as therapeutic targets in a wide spectrum of solid tumours Journal of Pathology 232 1 4 15 10.1002/path.4297 24588013

[b29-tjb-49-07-811] Pruitt KD Harrow J Harte RA Wallin C Diekhans M 2009 The consensus coding sequence (CCDS) project: Identifying a common protein-coding gene set for the human and mouse genomes Genome Research 19 7 1316 23 10.1101/gr.080531.108 19498102 PMC2704439

[b30-tjb-49-07-811] Qin A Johnson A Ross JS Miller VA Ali SM 2019 Detection of known and novel FGFR fusions in non–small cell lung cancer by comprehensive genomic profiling Journal of Thoracic Oncology 14 1 54 62 10.1016/j.jtho.2018.09.014 30267839

[b31-tjb-49-07-811] Ross JS Wang K Gay L Al-Rohil R Rand JV 2014 New routes to targeted therapy of intrahepatic cholangiocarcinomas revealed by next-generation sequencing The Oncologist 19 3 235 42 10.1634/theoncologist.2013-0352 24563076 PMC3958461

[b32-tjb-49-07-811] Servetto A Kollipara R Formisano L Lin CC Lee KM 2021 Nuclear FGFR1 regulates gene transcription and promotes antiestrogen resistance in ER+ breast cancer Clinical Cancer Research 27 15 4379 96 10.1158/1078-0432.CCR-20-3905 34011560 PMC8338892

[b33-tjb-49-07-811] Silverman IM Li M Murugesan K Krook MA Javle MM 2022 Validation and characterization of FGFR2 rearrangements in cholangiocarcinoma with comprehensive genomic profiling The Journal of Molecular Diagnostics 24 4 351 64 10.1016/j.jmoldx.2021.12.012 35176488

[b34-tjb-49-07-811] Simm D Hatje K Kollmar M 2014 Waggawagga: comparative visualization of coiled-coil predictions and detection of stable single α-helices (SAH domains) Bioinformatics 31 5 767 9 10.1093/bioinformatics/btu700 25338722

[b35-tjb-49-07-811] Speir ML Bhaduri A Markov NS Moreno P Nowakowski TJ 2021 UCSC Cell Browser: visualize your single-cell data Bioinformatics 37 23 4578 80 10.1093/bioinformatics/btab503 34244710 PMC8652023

[b36-tjb-49-07-811] Tate JG Bamford S Jubb HC Sondka Z Beare DM 2019 COSMIC: The catalogue of somatic mutations in cancer Nucleic Acids Research 47 D1 D941 7 10.1093/nar/gky1015 30371878 PMC6323903

[b37-tjb-49-07-811] Tuna M Amos CI Mills GB 2019 Molecular mechanisms and pathobiology of oncogenic fusion transcripts in epithelial tumors Oncotarget 10 21 2095 10.18632/oncotarget.26777 31007851 PMC6459343

[b38-tjb-49-07-811] Uguen A De Braekeleer M 2016 ROS1 fusions in cancer: A review Future Oncology 12 16 1911 28 10.2217/fon-2016-0050 27256160

[b39-tjb-49-07-811] Varadi M Bertoni D Magana P Paramval U Pidruchna I 2024 AlphaFold Protein Structure Database in 2024: providing structure coverage for over 214 million protein sequences Nucleic Acids Research 52 D1 D368 75 10.1093/nar/gkad1011 37933859 PMC10767828

[b40-tjb-49-07-811] Varadi M Nair S Sillitoe I Tauriello G Anyango S 2022 3D-Beacons: decreasing the gap between protein sequences and structures through a federated network of protein structure data resources GigaScience 11 giac118 10.1093/gigascience/giac118 36448847 PMC9709962

[b41-tjb-49-07-811] Wang Y Wang J Zeng T Qi J 2024 Data-mining-based biomarker evaluation and experimental validation of SHTN1 for bladder cancer Cancer Genetics 288 43 53 10.1016/j.cancergen.2024.09.002 39260052

[b42-tjb-49-07-811] Wiesner T He J Yelensky R Esteve-Puig R Botton T 2014 Kinase fusions are frequent in Spitz tumours and spitzoid melanomas Nature Communications 5 1 3116 10.1038/ncomms4116 PMC4084638 24445538

[b43-tjb-49-07-811] Wood CW Woolfson DN 2018 CCBuilder 2.0: Powerful and accessible coiled-coil modeling Protein Science 27 1 103 11 10.1002/pro.3279 28836317 PMC5734305

[b44-tjb-49-07-811] Wu EL Cheng X Jo S Rui H Song KC 2014 CHARMM-GUI membrane builder toward realistic biological membrane simulations Journal of Computational Chemistry 35 27 1997 2004 10.1002/jcc.23702 25130509 PMC4165794

[b45-tjb-49-07-811] Zhang M Ergin V Lin L Stork C Chen L 2019 Axonogenesis is coordinated by neuron-specific alternative splicing programming and splicing regulator PTBP2 Neuron 101 4 690 706 10.1016/j.neuron.2019.01.022 30733148 PMC6474845

[b46-tjb-49-07-811] Zheng S 2020 Alternative splicing programming of axon formation Wiley Interdisciplinary Reviews: RNA 11 4 e1585 10.1002/wrna.1585 31922356 PMC7594648

